# Transcranial Magnetic Stimulation Reveals Attentional Feedback to Area V1 during Serial Visual Search

**DOI:** 10.1371/journal.pone.0019712

**Published:** 2011-05-17

**Authors:** Laura Dugué, Philippe Marque, Rufin VanRullen

**Affiliations:** 1 Université Paul Sabatier, Toulouse, France; 2 Centre de Recherche Cerveau et Cognition, CNRS, UMR5549, Faculté de Médecine de Purpan, Toulouse, France; 3 Médecine Physique et de réadaptation, CHU Rangueil, Toulouse, France; Ecole Polytechnique Federale de Lausanne, Switzerland

## Abstract

Visual search tasks have been used to understand how, where and when attention influences visual processing. Current theories suggest the involvement of a high-level “saliency map” that selects a candidate location to focus attentional resources. For a parallel (or “pop-out”) task, the first chosen location is systematically the target, but for a serial (or “difficult”) task, the system may cycle on a few distractors before finally focusing on the target. This implies that attentional effects upon early visual areas, involving feedback from higher areas, should be visible at longer latencies during serial search. A previous study from Juan & Walsh (2003) had used Transcranial Magnetic Stimulation (TMS) to support this conclusion; however, only a few post-stimulus delays were compared, and no control TMS location was used. Here we applied TMS double-pulses (sub-threshold) to induce a transient inhibition of area V1 at every post-stimulus delay between 100 ms and 500 ms (50 ms steps). The search array was presented either at the location affected by the TMS pulses (previously identified by applying several pulses at supra-threshold intensity to induce phosphene perception), or in the opposite hemifield, which served as a retinotopically-defined control location. Two search tasks were used: a parallel (+ among Ls) and a serial one (T among Ls). TMS specifically impaired the serial, but not the parallel search. We highlight an involvement of V1 in serial search 300 ms after the onset; conversely, V1 did not contribute to parallel search at delays beyond 100 ms. This study supports the idea that serial search differs from parallel search by the presence of additional cycles of a select-and-focus iterative loop between V1 and higher-level areas.

## Introduction

For more than thirty years, and in particular since the precursory studies by Treisman and Gelade (1980) [1], visual search experiments have been used to study attention [1]–[4]. Visual search tasks consist in finding a target embedded among a number of distractor items. Reaction times (RT), and their variation as a function of set size (the total number of items present), are generally used to measure the influence of attention. A large amount of studies have allowed distinguishing between two kinds of visual search: parallel or “easy” search, with RT x set size slopes near zero msec/item, and serial or “difficult” search, with positive RT x set size slopes [5]. Despite some disagreements concerning the nature of attentional mechanisms [2], [6]–[8], it is generally accepted that parallel tasks reflect pre-attentive processing whereas serial tasks specifically involve attention [1], [2], [6]. Treisman proposed an influential framework for understanding the contribution of attention to serial visual search [1], [9], [10]: a master location map situated at the core of the attentional system would permit the selection of the target among the distractors. To summarize the principal features of this framework, visual inputs first reach visual areas via an early bottom-up wave within the first 100 ms or so after stimulus onset [11]–[13]. At this stage, visual areas decompose the visual input into separate feature maps (colour, orientation, motion…). Secondly, all of these signals converge towards higher level areas (e.g. FEF, PEF, PPC…) [10], [14], [15] to a “master location map” (also called a “saliency map” [15]) which selects the position of the most salient object. Finally, attention is focused by sending feedback projections to the feature maps at the selected location, allowing the concentration of neuronal resources on the selected object. If the selected location does not correspond to the target, the system iterates by selecting the next most salient location and re-focusing attentional resources, until the target is found. Interestingly, in this model the difference between parallel and serial tasks has a physiological correlate in the feedback projections from high-level areas, redistributing the contents of the master location map to low-level areas and feature maps. This interaction between higher-level and lower-level areas should be persistently active during serial search until the target is found, whereas for parallel search it should vanish quickly after stimulus onset (since finding the target would only require a single feed-forward pass through the system). Thus, at the level of early visual areas, the influence of attention (i.e. the result of the feedback projections) should be visible at longer post-stimulus latencies for serial search compared to parallel search tasks. The present study was specifically designed to test this hypothesis using Transcranial Magnetic Stimulation (TMS).

TMS is an experimental tool that creates interference with specific neural processes, precisely in space and time [16], [17]. Over the last ten years, TMS has often been used to understand the role of high-level areas, such as FEF or PPC [18]–[21], during serial versus parallel visual search tasks. In terms of the framework introduced above, these studies were interested in probing the high-level areas supporting the saliency map itself. Thus, authors have demonstrated that FEF and PPC, notably, are involved in serial tasks where the target is defined as a conjunction of simple features, but not in parallel tasks where a single elementary feature defines the target [22]. Others showed that FEF is involved before PPC during conjunction search [19]. These data support the notion of a saliency map in the higher levels of the visual system hierarchy. Another important aspect of the attention system, which has been less extensively explored so far, is the iterative feedback to lower-level areas. In particular, due to its retinotopic organization, targetting the primary visual area V1 in a TMS study can allow for a specific control of the spatial correspondence between the affected cortical location and the position of the search array within the visual field.

Currently, Juan and Walsh (2003) [23] are the only ones who studied the role of V1 during visual search using TMS. In a first experiment, they demonstrated that repetitive TMS (rTMS) applied on V1 within the first 100 ms after stimulus onset disrupts both parallel and serial visual search tasks; however, if rTMS begins later than 100 ms after the onset, it only interferes with serial attentive visual search. Moreover, in a second experiment using double-pulses of TMS (40 ms interval) applied on V1 at different delays from the onset, they showed that the specific involvement of V1 in serial search was restricted to a particular latency (200–240 ms) after the onset of the search array. Overall, this study provided arguments for a late involvement of visual attention in V1 during serial visual search, compatible with the idea of an attentional feedback from higher-level areas. However, in that study the TMS interference was evaluated against a baseline performance obtained without any TMS; furthermore, no specific care was taken to present the search array at the retinotopic location corresponding to the stimulated cortical region. It remains possible, therefore, that the reported interference actually reflected a non-specific distraction induced by the auditory “click” or the somatosensory “tap” accompanying the TMS pulses [24], [25], rather than the postulated attentional feedback; this distraction may have been more detrimental during the serial search, due to the more difficult nature of the task.

The goal of our study was thus to confirm the conclusions of Juan and Walsh (2003) [23], by comparing the application of TMS pulses on V1 at various post-stimulus delays within vs. away from the retinotopic location of the stimulated cortical site. We reasoned that this procedure, better than a classic “sham” stimulation [26]–[29], would allow us to identify and discard any non-specific effects of the stimulation (e.g. distraction induced by the “click” and “tap” accompanying each pulse). Information about the visual scene activates low-level areas in a feedforward manner within 40 to 100 ms [11]–[13]. Consequently, we expected that a magnetic pulse applied to V1 in the first 100 ms post-stimulus would interfere equally with both parallel and serial visual search tasks and thus we did not test these delays. However, for longer delays we expected that TMS to V1 should specifically alter serial visual search, because of the need for feedback signals from higher-level areas to focus attention on the target. Finally, any attentional impairment caused by TMS would need to be restricted to stimulation of the cortical region matching the retinotopic location of the search array; any non-specific impairment, on the other hand, could be attributed to distraction or other motivational factors (e.g., stress or anxiety in anticipation of the pulses).

## Results

In a preliminary experiment on the same group of subjects (n = 12) that also participated in the main experiment, we determined search slopes (that is, the variation in reaction time as a function of set size) for the two tasks that we intended to use in the main experiment: finding the letter T among Ls, and finding the symbol + among Ls. Targets and distractors were randomly and independently rotated. On each trial the set size varied randomly between 4 and 8 elements. As expected, we found that subjects employed a serial search strategy for the former task (slopes 33.8 ms±10.9 ms per item) and a parallel strategy for the latter (slopes 1.6 ms±0.8 ms per item).

**Figure 1:**
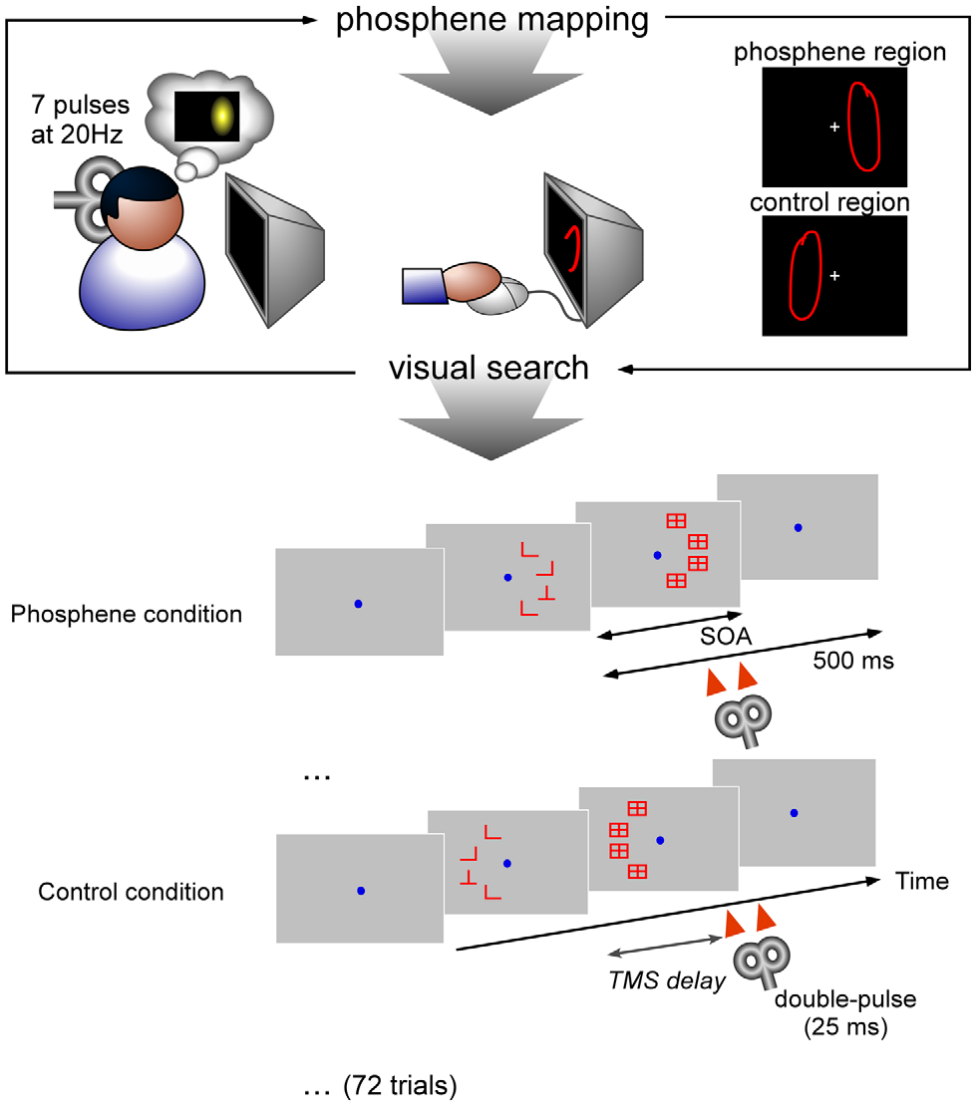
Experimental protocol. The main experiment was divided in two parts. The first part consisted in phosphene mapping. The subjects received 7 magnetic pulses at 70% output intensity. They were then asked to draw on the screen the perceived phosphene using the mouse. This area, which we called the “phosphene region”, was during the second part –the visual search experiment– the region where the stimuli were presented. This ensured that the TMS effect was specific to the stimulated area on the cortex. The control used was the symmetric zone relative to the vertical midline, which we called the “control region”. The visual search experiment comprised blocks of either a serial task (L versus T) or a parallel task (L versus +). The SOA (Stimulus Onset Asynchrony) between the search array and the masks was adjusted individually in each task to fix performance at around 75%. A double-pulse (25 ms interval) was applied at random delays between 100 and 450 ms after the onset (8 different delays by steps of 50 ms). Every 72 trials (2 blocks of 36 trials), the experiment restarted from the first step to make sure that the phosphene had not changed.

In the main experiment, we analyzed the effects of administrating sub-threshold TMS double-pulses over the primary visual cortex, at 8 different post-stimulus delays from 100 ms to 450 ms (50 ms steps), for the serial (L vs. T) and the parallel (L vs. +) search tasks. To this aim, we considered two conditions. In the “phosphene region” condition the stimuli were placed at the same screen location where the subjects had previously seen and drawn a phosphene (an illusory light pattern) upon application of supra-threshold intensity TMS over the same cortical site. In the “control region” condition the stimuli were placed at the opposite screen location compared to the vertical midline (figure 1) corresponding to a retinotopically-defined control location. The search arrays were displayed for a certain duration (SOA) determined for each subject to obtain 75% correct performance, and then masked to reach a total trial duration of 500 ms. For this experiment the set size was fixed at 4 items, and only the presence or absence of the target varied randomly on each trial. Every 72 trials the experiment was paused and the phosphene position was re-tested to ensure that it had not moved (if it had, the new phosphene location was recorded and used for the following 72 trials). 900 trials were performed in each task by each of the 12 subjects. As we expected TMS to interfere specifically with the detection of the target, performance was analyzed in terms of hit rates. (No significant effect was found when analyzing false alarm rates).

**Figure 2:**
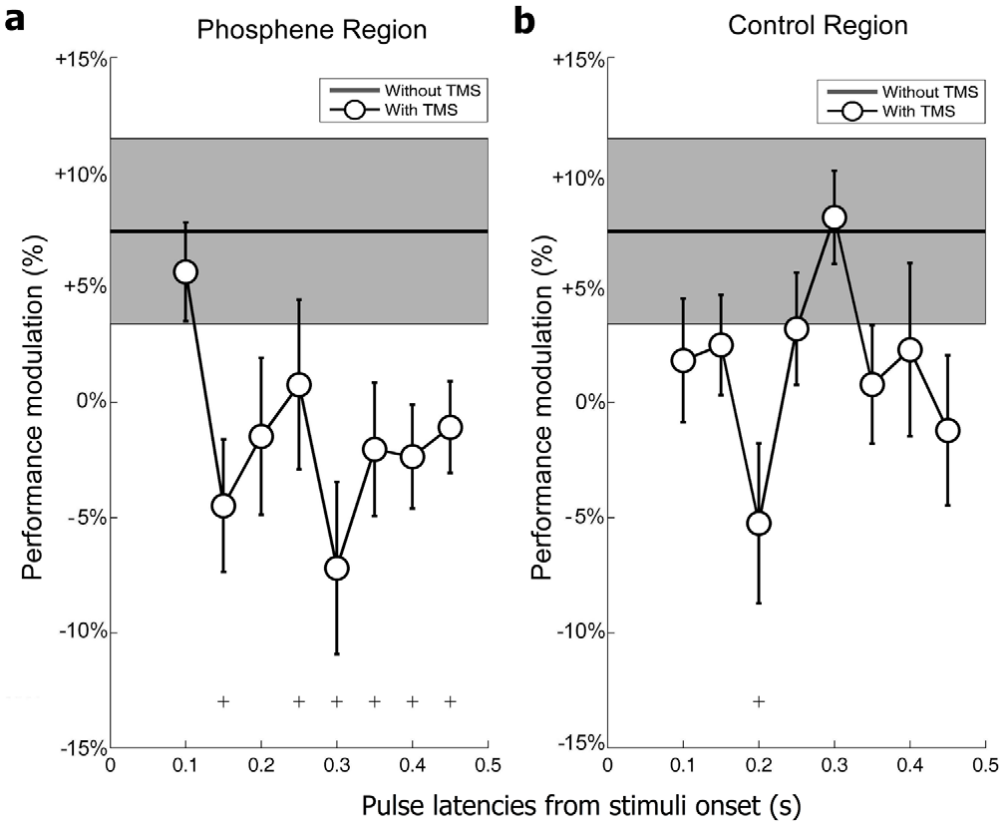
TMS latency effects on serial search (L versus T). The variations in performance are plotted as a function of pulse latency. The zero baseline corresponds to the mean hit rate across both TMS conditions (phosphene and control regions) and across all pulse latencies. Error bars represent standard error of the mean (SEM). The condition “without TMS” (also normalized with respect to the same baseline) is indicated by the horizontal line and shaded area (mean ± SEM); the hit rate without TMS was about 7% higher on average than in the TMS conditions. The symbol ‘+’ denotes a significant difference (paired t-test, p<0.05) between the hit rate observed at a given pulse latency and the hit rate without TMS (these differences did not remain significant after Bonferroni's correction for multiple comparisons). **a.** Mean of 12 subjects' hit rates in the serial task over the “phosphene region” condition. Lower hit rates than in the no-TMS condition are observed at 6 pulse latencies, including one cluster of 5 consecutive pulse latencies (250–450 ms). A cluster analysis based on a bootstrapping procedure demonstrates that the presence of a significant performance decrease for 5 consecutive latencies is unlikely to be due to chance (p<0.05). **b.** Mean of 12 subjects' hit rates in the serial task, over the “control region” condition. Only one pulse latency (200 ms) generated a significant difference between the TMS and no TMS conditions. No cluster was significant.

First, we compared performance following TMS double-pulses to a baseline condition without any TMS (Figure 2). In order to render performance comparable across subjects, the mean hit rate across all TMS conditions (phosphene and control regions, at all delays) was subtracted from all hit rate values (including those for the condition without TMS). For the serial search task (L vs. T), paired t-tests (one test at each pulse latency) showed significant differences (p<0.05, uncorrected; these differences were not significant after Bonferroni's correction for multiple comparisons was applied) between the TMS condition and the no-TMS condition, both in the “phosphene region” (figure 2a) and in the “control region” (figure 2b). That a TMS double-pulse can generate a performance decrement for stimuli presented far outside of the retinotopic region directly affected by the TMS, is most certainly an indication of a non-specific bias, e.g. due to the auditory or somato-sensory distractions accompanying the pulses. The decrements in search performance were more sustained, however, in the phosphene region, which counted 5 consecutive significant points (compared to only one point for the control region). A cluster analysis was designed, in which the performance during the TMS condition was exchanged with the performance during the no-TMS condition for a random subset of subjects, and the t-tests were subsequently recomputed. Each time (4095 repetitions corresponding to all 2^12^ possible subject combinations, except for the actual dataset), the maximum number of consecutive significant latencies in the surrogate data was compared to the observed cluster size of 5 consecutive significant latencies. This analysis revealed that the steady decrease in search performance following TMS pulses applied between 250 and 450 ms post-stimulus was unlikely to occur by chance (p = 0.0232). On the other hand, the same cluster analysis did not reveal a significant performance decrease in the “control region” condition. Finally, in order to complement these results a two-way Anova (pulse latency X presentation zone) was performed. It showed a main effect of the presentation region (F(1,176) = 4.39, p = 0.0376) but no main effect of latency (F(7,176) = 1.09, p = 0.372). Importantly, it also revealed a significant interaction between the two factors (F(7,176) = 2.3, p = 0.029), implying that TMS exerted its effects more strongly at specific latencies in the “phosphene region” condition.

**Figure 3:**
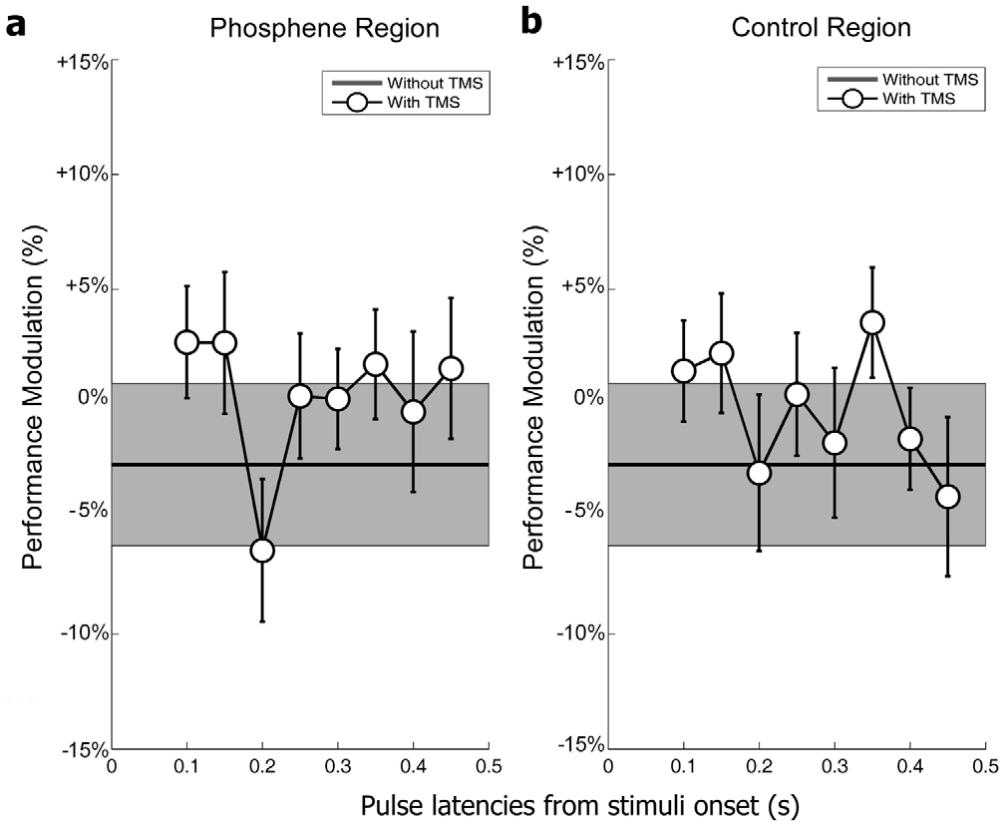
TMS latency effects on parallel search (L versus +). Plotting conventions are similar to those in Figure 2. **a.** Mean of 12 subjects' hit rates in the parallel task, over the “phosphene region” condition. There is no significant decrease of performance relative to the no-TMS condition. **b.** Mean of 12 subjects' hit rates in the parallel task, over the “control region” condition. There is no significant decrease of performance relative to the no-TMS condition.

The same analyses were performed for the parallel task (figure 3). In this case, no significant difference compared to the baseline was observed in either region condition, regardless of pulse latency (paired t-tests, p>0.05). Similarly, the two-way Anova showed no significant main effect of stimulation region (F(1,176) = 0.2, p = 0.6527) or pulse latency (F(7,176) = 1.5, p = 0.1687), nor any interaction between the two (F(7,176) = 0.44, p = 0.8757).

**Figure 4:**
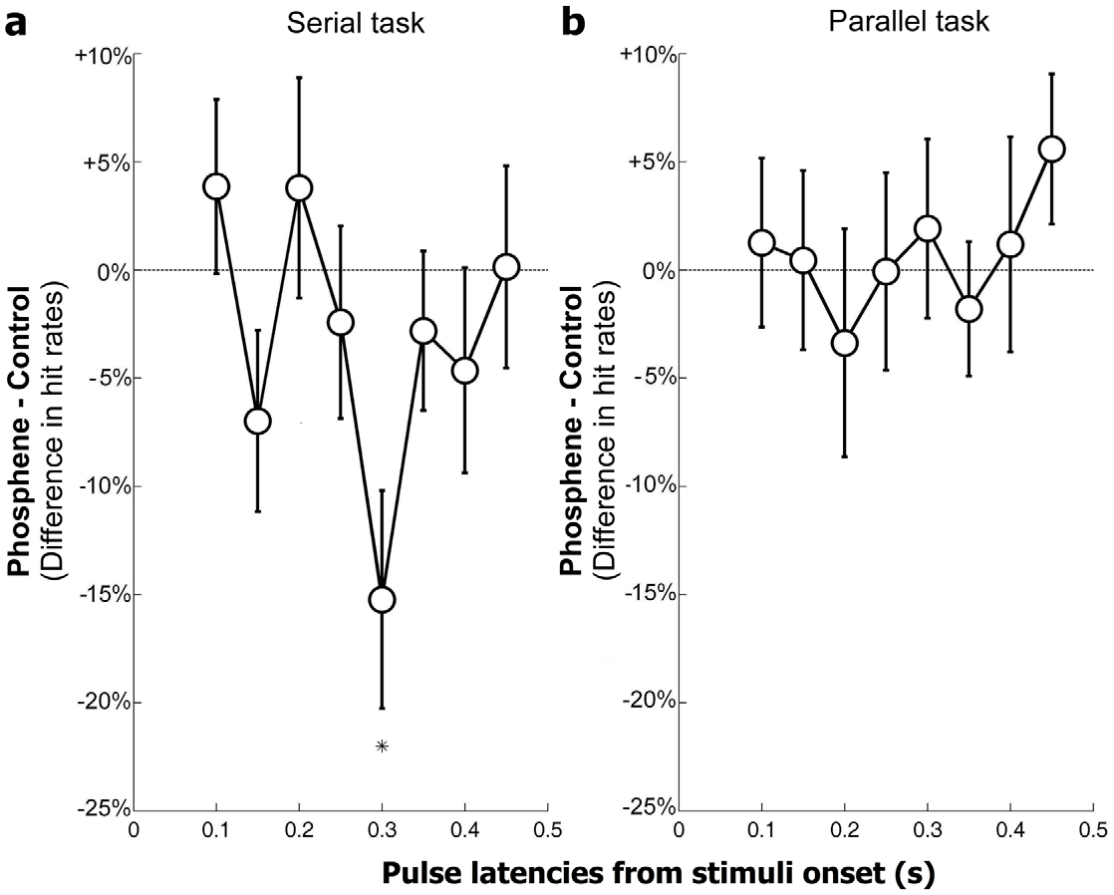
TMS effects on visual search (phosphene region vs. control region). These graphs represent the difference in hit rates between trials where the stimuli appeared in the phosphene region and those where they appeared in the control region. Error bars represent Standard Error of the Mean (SEM). The symbol ‘*’ indicates significant differences (t-test, p<0.05, Bonferroni-corrected for multiple comparisons). **a.** Mean of 12 subjects' hit rates differences for the serial task. There is a significant decrease in performance when the double-pulse is applied at a delay of 300 ms (t(11) = 3.03, p = 0.0057). This means that hit rates are significantly lower in the specific phosphene region than in the control region. **b.** Mean of 12 subjects' hit rates differences for the parallel task. There is no significant difference.

To summarize these results, we computed the difference of hit rates between trials for the “phosphene region” condition and the “control region” condition at each pulse latency (Figure 4). In the case of the serial task (figure 4a.), the difference was significantly lower than zero (the baseline, indicating no difference between phosphene and control regions) at a specific delay of 300 ms (t = −3.03, df = 11, p = 0.0057). This difference remained significant (p<0.05) after Bonferroni's correction for multiple comparisons. Looking back on the hit rates obtained in each region, this significant difference at 300 ms appears to correspond both to a TMS-induced performance decrease in the phosphene region, and to a concurrent relative *increase* in the control region. While counterintuitive, this increase could in fact be explained by a competitive process, such that when TMS inhibits attentional deployment in the (contralateral) phosphene region, it simultaneously facilitates deployment in the symmetric (ipsilateral) region, thus inducing an increase in performance. Finally, there was no significant difference between regions for the parallel task (figure 4b). For both tasks, a one-way Anova did not demonstrate any significant difference in hit rates between delays (F(7,88) = 1.91, p = 0.0777 for serial task ; F(7,88) = 0.39, p = 0.9055 for parallel task).

## Discussion

One notable aspect of our results is that the administration of TMS over occipital cortex induced non-specific biases that could easily have been mistaken for attentional effects. Indeed, especially in the serial search task, we observed significant differences for certain delays of double pulse of TMS application, compared to the no-TMS condition. The fact that this effect was also present when the visual stimuli were presented outside of the retinotopic region directly affected by TMS (“control region” condition) indicates that the effect was not related to the visual task at hand. Instead, as explained in the Introduction, it can be assumed that the sensation on the skin and/or the auditory noise accompanying the pulses could have distracted subjects and caused a disruption of performance [24], [25]. Interestingly this non-specific effect was only found in the serial, but not in the parallel task. This difference could be attributed to the complexity of the serial task which may have rendered observers more sensitive to external disturbances. The existence of such non-specific TMS effects implies that a mere difference between TMS and no-TMS conditions cannot be taken to reflect direct interference with visual or attentional processes, and that further control measures are necessary to reach meaningful conclusions. Our TMS study is the first to address the contribution of V1 to visual search processes while using such a control: here, the presentation of visual stimuli in a non-relevant retinotopic region relative to the site of stimulation on the occipital cortex. This procedure allowed us to isolate a specific delay for TMS interference over the occipital cortex. The administration of a double pulse of TMS over the primary visual area ∼300 ms after stimulus onset specifically impaired subjects' performance in a serial, attentional visual search task whereas there was no effect in a parallel visual search task. Consequently, under the particular conditions of our experiments (i.e., using Ls and Ts as stimuli, and with a set size of 4 elements), V1 contributed to attentional selection of the visual search target at a specific delay of ∼300 ms after stimulus onset. The differences and similarities between our results and those of the previous study by Juan and Walsh (2003) are listed in Table 1.

**Table 1:** Comparison between the methods and results of Juan & Walsh (2003) and those of the present study.

		Juan & Walsh (2003)	present study
Paradigm	Parallel search task	Blue circle amongst red circles (or vice-versa)	L vs. +
	Serial search task	Blue/amongst blue\and red/	L vs. T
	TMS delays<100 ms	Yes	No
	TMS delays>100 ms	3 delays (140, 200, 260 ms)	8 delays (100, 150, 200, 250, 300, 350, 400, 450 ms)
	Double-pulse interval	40 ms	25 ms
	Control condition	no TMS	Retinotopically specific presentation
Results	TMS effects<100 ms	Yes (parallel task)	Not tested
	TMS effects>100 ms	200 ms (serial task)	300 ms (serial task)
	Non-specific TMS effects	Not tested	∼200 ms (serial task)

“Non-specific TMS effects” refers to TMS pulses affecting visual performance even outside of the stimulated retinotopic region. Delay values correspond to the first of the double-pulse.

The present results can be easily interpreted within the framework described in the Introduction. For a parallel visual search the target can be selected after a single feed-forward sweep (a “pop-out” effect), and V1's contribution will only be visible early after stimulus onset. Indeed, while Juan & Walsh (2003) reported specific TMS interference on V1 within 100 ms after the onset, our study did not reveal any such interference for a parallel task at post-stimulus delays between 100 ms and 500 ms. The serial visual search task, on the other hand, requires iterative attentional selection of the target via feedback from higher-level areas implementing the “saliency map”. With our stimuli and a set size of 4 elements, this feedback exerted its strongest effect on V1 at post-stimulus delays around 300 ms. This framework further predicts that increasing the set size to say, 8 elements, should lead to TMS interference at longer post-stimulus delays in the serial task (because more iterations of the “select-and-focus-attention” loop will be needed on average), while no difference should occur in the parallel task (because the target always pops out within the first feed-forward sweep, regardless of set size). Further experiments will hopefully confirm this prediction.

## Materials and Methods

### Subjects and Ethics Statement

The participants were aged 20–35 years. Overall, 15 different subjects participated in the experiments, 6 females and 9 males. 11 of these took part in both tasks. 13 subjects participated in the serial task (L versus T) and 13 subjects participated in the parallel task (L versus +). One subject who participated to both experiments was excluded from the analysis because his results were considered as “outliers” (hit rates on the serial task were more than 2.5 standard deviations away from the group average). In the end, data from 12 subjects were analyzed in each of the two tasks. All participants gave written informed consent prior to taking part in the experiment. Standard exclusion criteria for TMS were applied [24], [25]. The study was approved by the local ethics committee “CPP Sud-Ouest et Outre-Mer I” under protocol number 2009-A01087-50.

### Apparatus and stimuli

Subjects were placed 57 cm from the screen, which measured 36.5°×27° of visual angle. Their head was maintained in a fixed position using a chinrest and headrest in front of them, as well as a 70 mm figure-of-eight coil that was pressed against the subjects' scalp in the occipital region. Magnetic stimulations were applied with a Magstim Rapid_2_ stimulator of 3.5 Tesla, which produces a biphasic current. The stimuli were presented on a uniform gray background.

Two kinds of tasks were performed: a serial and a parallel visual search tasks. Subjects reported the presence or absence of a target which could be either a “T”, for the serial task, or a “+” for the parallel task, among distractors, which were in both cases “L”s. On each trial there were either 4 distractors or 3 distractors and 1 target. (We have checked in a preliminary experiment with variable set sizes between 4 and 8 elements that each subject used a serial strategy for the L versus T task, with positive RT x set size slopes, and a parallel strategy for the L versus + task, with near zero RT x set size slopes). Each letter could be presented randomly in 4 orientations: 0°, 90°, 180° and 270° from upright. The target was present in half of the trials, randomly determined.

The subject initiated a trial by pressing a button. 1.5 to 2.5 seconds later, the stimuli appeared and then disappeared after a certain SOA (Stimulus-mask Onset Asynchrony), replaced by visual masks. SOAs were predefined for each subject to achieve about 75% correct. The mean of the subjects' SOA for the serial task was 144.62 ms±57 ms, and 63.07 ms±20 ms for the parallel visual search. The total trial duration (stimulus+mask presentation) was 500 ms.

Subjects were asked to respond accurately without any time pressure whether the target was present or absent by pressing a button on the computer's keyboard. We then analysed the data in terms of hit rates [30], since we expected that TMS at certain latencies could specifically affect the target detection.

### Procedure

#### Determination of the presentation zone

Subjects were placed in the dark and kept their gaze on a dim fixation point at the center of the screen. 7 pulses at 70% of intensity were applied during 300 ms (i.e. at 20 Hz) with the coil placed over the supposed V1 region of the scalp, 1 cm above the inion. The stimulations were applied either on the left or the right hemisphere (2 cm away from the midline) to induce phosphenes either in the right visual field or in the left. Subjects were then asked to draw the phosphene they had seen as precisely as possible. They were given an opportunity to repeat the procedure and verify the location and shape of the phosphene, until they were satisfied with their response. This zone, which we call the “phosphene region”, will be in the second part of the experiment the zone of stimulus presentation. We used the symmetrical region of this phosphene zone, relative to the vertical midline, as a “control region” for stimulus presentation (we expected that TMS should not affect the processing of stimuli presented in this zone).

#### Visual search tasks and dTMS

Subjects performed 25 blocks of 36 trials (figure 1). There were two conditions of stimulus presentation in each block. In half of the trials stimuli were presented in the “phosphene region”, and in the other half they were presented in the “control region”. Half of the blocks had the phosphene region in the left visual field, and half in the right. For this part of the experiment the magnetic stimulation was applied at sub-threshold intensity; consequently subjects did not perceive phosphenes during the stimulus presentation. The stimuli were always at a constant eccentricity to the central fixation point. Every two blocks, we checked that the phosphene region had not moved or changed its shape (see above).

During the visual search experiment, subjects received one double-pulse of TMS in each trial, in a procedure similar to that used in the Juan and Walsh article (2003) [23] but with some adjustments. There was no pulse in the first 100 ms because we hypothesized that such early latencies should correspond to the first wave of activation of visual areas, common to both tasks, and should thus remain immune to attentional feed-back effects. We then presented the double-pulse at 8 different delays from 100 to 450 ms, every 50 ms (100, 150, 200, 250, 300, 350, 400, 450 ms). The interval between the two TMS pulses was 25 ms, and the “latency” refers to the first one of these pulses. Finally, the subjects performed two more blocks (randomly interleaved) without magnetic stimulation, to test whether the presence of a pulse (sensation, noise…) could induce a non-specific bias. This no-TMS condition was used as our baseline.

### Statistical analysis

Visual search data in each task were analyzed using Anovas with two independent variables: the pulse delay, and the presentation zone. We tested if these variables could influence subject performance; the dependent variable was the hit rate. To minimize irrelevant differences between subjects visual search abilities, a normalization was applied by subtracting from their hit rate in each region condition (phosphene and control regions) the average of the hit rates on both conditions. The hit rate differences compared to this baseline were represented as a modulation of performance (in % of modulation). Paired t-tests were also used to compare subject performances when they received a double-pulse of TMS at a given delay with the condition without any pulse. The α significance level was set at 5%. Whenever a significant effect was found, we reported the effect, and also evaluated and reported whether its significance was robust to a correction for multiple comparisons across delays using Bonferroni's method. Finally, in the “phosphene region” condition for the serial task, a cluster analysis was performed, using a permutation test (exchanging for a random subset of subjects the hit rates of the TMS condition with the hit rates of the no-TMS condition and recomputing the paired t-tests, then repeating the procedure several times) to determine the probability of obtaining by chance 5 consecutive, significant points. 4096 iterations were computed corresponding to all the possible combinations of subjects (2^12^, 12 corresponding to the number of subjects) and the α significance level was set at 5%, that is to say less than 204 iterations resulting in the occurrence of a cluster of 5 consecutive, significant points.
